# DNA Methylation of *TLR4, VEGFA*, and *DEFA5* Is Associated With Necrotizing Enterocolitis in Preterm Infants

**DOI:** 10.3389/fped.2021.630817

**Published:** 2021-03-04

**Authors:** Daphne H. Klerk, Torsten Plösch, Rikst Nynke Verkaik-Schakel, Jan B. F. Hulscher, Elisabeth M. W. Kooi, Arend F. Bos

**Affiliations:** ^1^Division of Neonatology, Beatrix Children's Hospital, University Medical Center Groningen, University of Groningen, Groningen, Netherlands; ^2^Department of Obstetrics and Gynecology, University Medical Center Groningen, University of Groningen, Groningen, Netherlands; ^3^Division of Pediatric Surgery, Department of Surgery, University Medical Center Groningen, University of Groningen, Groningen, Netherlands

**Keywords:** neonatology, DNA methylation, epigenetics, preterm infant, necrotizing enterocolitis, Toll-like receptor 4, vascular endothelial growth factor A, defensin alpha 5

## Abstract

**Background:** Epigenetic changes, such as DNA methylation, may contribute to an increased susceptibility for developing necrotizing enterocolitis (NEC) in preterm infants. We assessed DNA methylation in five NEC-associated genes, selected from literature: *EPO, VEGFA, ENOS, DEFA5*, and *TLR4* in infants with NEC and controls.

**Methods:** Observational cohort study including 24 preterm infants who developed NEC (≥Bell Stage IIA) and 45 matched controls. DNA was isolated from stool samples and methylation measured using pyrosequencing. We investigated differences in methylation prior to NEC compared with controls. Next, in NEC infants, we investigated methylation patterns long before, a short time before NEC onset, and after NEC.

**Results:** Prior to NEC, only *TLR4* CpG 2 methylation was increased in NEC infants (median = 75.4%, IQR = 71.3–83.8%) versus controls (median = 69.0%, IQR = 64.5–77.4%, *p* = 0.025). In NEC infants, *VEGFA* CpG 3 methylation was 0.8% long before NEC, increasing to 1.8% a short time before NEC and 2.0% after NEC (*p* = 0.011; *p* = 0.021, respectively). A similar pattern was found in *DEFA5* CpG 1, which increased from 75.4 to 81.4% and remained 85.3% (*p* = 0.027; *p* = 0.019, respectively). These changes were not present for *EPO, ENOS*, and *TLR4*.

**Conclusion:** Epigenetic changes of *TLR4, VEGFA*, and *DEFA5* are present in NEC infants and can differ in relation to the time of NEC onset. Differences in DNA methylation of *TLR4, VEGFA*, and *DEFA5* may influence gene expression and increase the risk for developing NEC. This study also demonstrates the use of human DNA extraction from stool samples as a novel non-invasive method for exploring the bowel of preterm infants and which can also be used for necrotizing enterocolitis patients.

## Introduction

Necrotizing enterocolitis (NEC) is a severe gastrointestinal inflammatory disease that mainly affects preterm-born infants. The etiology of NEC is not completely understood but is most likely multifactorial, with prematurity having a central role (1). Four of the main components preceding NEC are the immature intestinal barrier, immature circulatory regulation of the intestines, abnormal motility patterns and an immature immune system. Other contributing factors are formula feeding, abnormal colonization by potentially pathogenic bacteria and hypoxic ischemic injury (1, 2).

However, when comparing preterm infants with a similar risk profile, some develop NEC while others do not (3). This variable susceptibility in some infants suggests that the current multifactorial model may not be complete (4). We hypothesize here that epigenetic alterations contribute to this predisposition of some infants for developing necrotizing enterocolitis. The study of epigenetics involves changes in gene expression that are not caused by modifications in the genetic code. Increasing evidence suggests epigenetic modifications to be the link between early environmental stressors and later disease (5). We know of several environmental risk factors for NEC, such as antibiotic exposure, formula feeds, gut microbiota alterations, prolonged gut hypoxia and red blood cell transfusions. Several of these have already been directly associated with epigenetic alterations. Microbiota alterations in particular can induce epigenetic changes in immature intestinal epithelial cells (6).

The best studied epigenetic mechanism is DNA methylation. Methylation usually occurs at cytosine-phosphate-guanosine (CpG) positions in the DNA. An increase of methylation in gene promoters is often associated with a decrease in gene expression. DNA methylation is dynamic and can change over time, in response to environmental stimuli (5). The fetal and neonatal period is known to be prone for changes in DNA methylation and many differentially methylated CpGs have been identified in preterm-born infants (7). Changes in DNA methylation of several percentages can already cause significant changes in gene expression. DNA methylation in preterm born infants with NEC has been evaluated only in surgically treated NEC patients. There, clear differences in the epigenome of NEC infants was found compared to controls, as well as an association between DNA methylation and gene expression (8). Preterm born pigs affected by NEC also show differences in DNA methylation of several intestinal genes, compared to unaffected preterm born pigs. The same study underlines the dynamic nature of DNA methylation of intestinal genes in the first day after birth, which is increased after exposing the immature intestine to formula feeding (9). Other differences in epigenetic mechanisms, for example dysregulated micro-RNAs, are present as well in NEC affected infants (10).

The above led us to hypothesize a potential role for DNA methylation in the development of NEC. We selected five genes after reviewing literature on methylation of intestinal wall derived genes previously reported to have a potential role in the development of NEC including erythropoietin (*EPO*) (11, 12), vascular endothelial growth factor A (*VEGFA*) (13–15), endothelial nitric oxide synthesis (*ENOS*) (16), defensin alpha 5 (*DEFA5*) (17), and toll-like receptor 4 (*TLR4*) (18–20).

*EPO, VEGFA*, and *ENOS* are involved in the perfusion of the intestine. *EPO* regulates the red blood cell production and prevents down-regulation of the intestinal tight-junction protein ZO-1 (11). Its gene product, erythropoietin, naturally occurs in breast milk, which reduces the risk of NEC development, especially in very low birth weight infants (12). Treatment with erythropoietin also lowered the incidence of NEC and tight junction damage in mouse pups (11). *VEGFA* promotes proliferation of endothelial cells, tube formation, and facilitates angiogenesis (13). In human and experimental NEC models, there is a decrease in the VEGFA protein in intestinal tissue (14). Genetic variations of *VEGF* that decrease *VEGF* expression increase the risk of NEC development (15). *ENOS* is responsible for the majority of nitric oxide (NO) production, the most potent vasodilator stimulus active in the new-born intestine. Expression of *ENOS* and production of NO is decreased in the arterioles of NEC infants (16).

*DEFA5* and *TLR4* are both important for the intestinal immunity. The gene product of *DEFA5* is an anti-microbial protein secreted by Paneth cells and *TLR4* belongs to the group of pattern recognition receptors. Both an increase and a decrease of immune competent Paneth cells and *DEFA5* expression has been suggested as a cause of NEC (17). *TLR4* is expressed on both immune and intestinal cells and can be upregulated in response to stress (18). *TLR4* is also required for the development of the gut, it is therefore highly expressed during fetal development. With the abundance of TLR4 receptors in the preterm gut, activation by LPS may cause an overwhelming inflammatory response. The release of inflammatory cytokines as well as an increase in enterocyte apoptosis and a reduction in enterocyte proliferation can lead to NEC (19). Small intestinal cells have a higher expression of *TLR4* when NEC is present in animal studies, compared to healthy intestinal cells (18, 20).

To test our hypothesis that methylation of these genes is indeed associated with NEC we first aimed to determine whether a difference between NEC affected infants and their non-affected peers existed in DNA methylation prior to NEC onset of the *EPO, VEGFA, ENOS, DEFA5*, and *TLR4* genes. Second, in the infants diagnosed with NEC, we aimed to investigate whether methylation patterns were different long before, a short time before and after NEC onset.

## Methods

**Figure 1:**
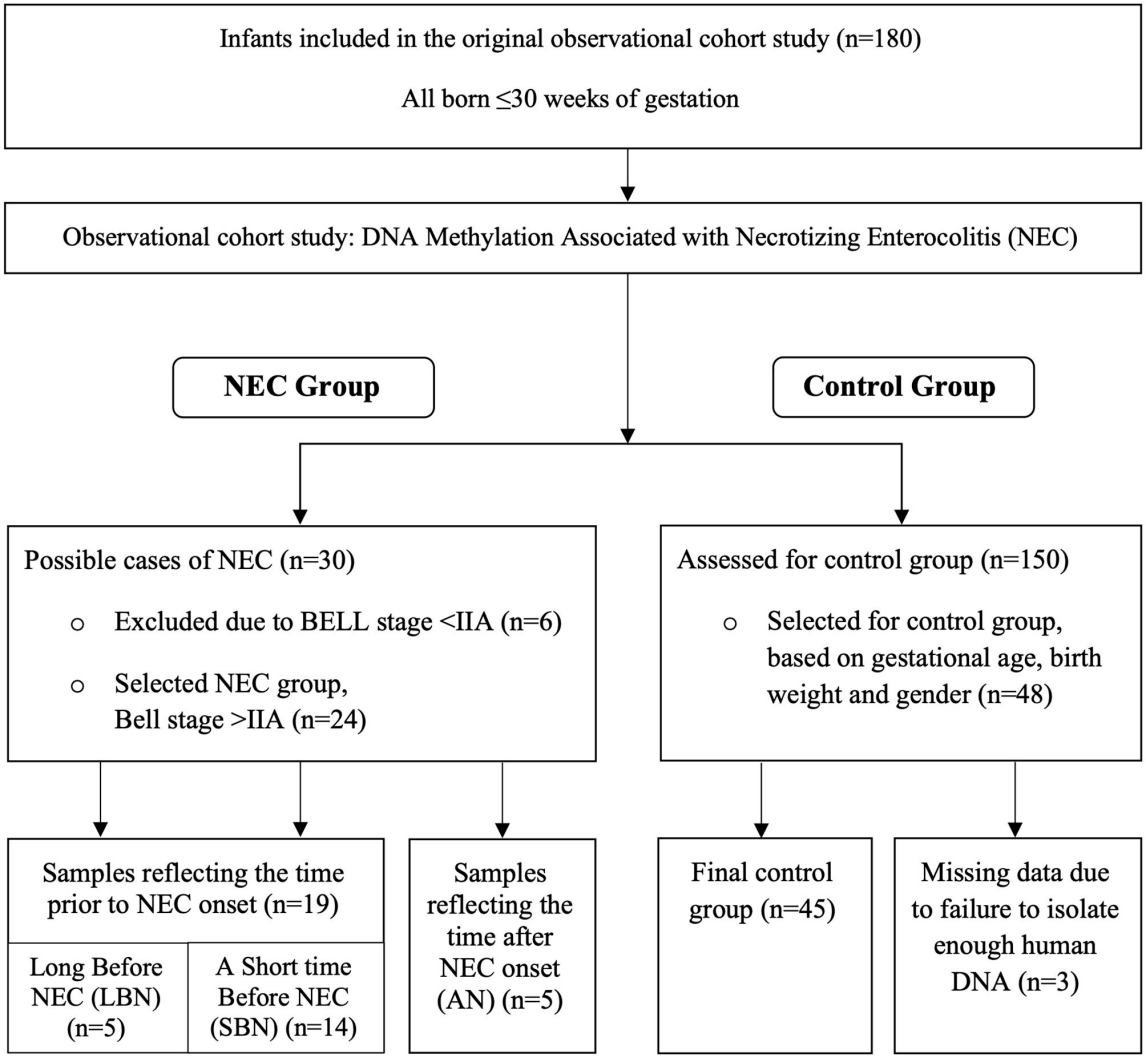
Flow diagram presenting the selection of Necrotizing Enterocolitis (NEC) infants and controls.

We performed an observational cohort study, with a nested case-control design. We included infants admitted to the level III Neonatal Intensive Care Unit of the University Medical Center Groningen (UMCG) from April 2016 to December 2018. For this pilot study, we used residual stool samples of an ongoing multicenter observational cohort study on fecal volatile organic compounds (VOCs), analyzed by an electronic nose (21). We included infants born after <30 weeks of gestation. Written informed consent was obtained from the parents and the Institutional Review Board of the UMCG approved the study. Using the residual samples for this study was permitted under the Dutch Medical Research with Human Subjects Law. Of all participating infants in the original cohort study, 24 developed definite NEC with Bell's stage 2A or higher. The diagnosis NEC was established via the presence of pneumatosis intestinalis and/or portal venous gas on the abdominal X-ray, or during surgery. Cases of SIP were therefore excluded on a clinical and pathological basis. Out of the remaining 150 infants, 48 were matched to the NEC affected infants. The primary matching criterium was gestational age, for which we accepted a difference of maximum 4 days. In case there were multiple candidates, similar birth weight and sex were used to select the controls. We excluded infants with suspected NEC (Bell's stage < II). In Figure 1 we display the subject selection.

### Fecal Sample Selection

We measured DNA methylation of *EPO, VEGFA, ENOS, DEFA5*, and *TLR4* by isolating human DNA from stool samples. Human DNA in stool samples mainly originates from intestinal epithelium cells. These cells renew every 4–5 days in adults and are shed into the feces, however this proliferation process may be increased in preterm infants (22). The main types of cells shed into the stool in infants are enterocytes, goblet cells, Paneth cells, and enteroendocrine cells, whereas only a small number of cells of lymphoid origin is present (23, 24). The amount of human DNA in stool samples is low, <1% of the total DNA, as the vast majority of DNA present in feces is bacterial DNA from the gut microbiota (25).

From every stool in the first 28 days after birth or until transfer to a level II hospital, samples were collected and stored at −20 degrees. The stool samples used for the current study were preferably selected from the days around onset of NEC, and in controls around a similar postnatal age. We collected samples at three different time points and defined three groups accordingly.

The first group (a Short time Before NEC, SBN) consisted of infants in which we selected samples from the week before NEC onset or until 2 days after NEC onset, depending on the availability of the samples. Samples taken in this window contain intestinal cells that are around 4–5 days old. They therefore reflect DNA methylation patterns which are present a short time before the onset of NEC. In the second group (Long Before NEC, LBN) we collected samples from infants who developed NEC after the first 28 days in which the stool samples were collected. For these infants, we selected the last collected sample for analysis. These samples reflect a somewhat longer time before NEC onset.

For the control infants, we matched stool samples based on postnatal day of the samples from the NEC affected infants. The stool samples from the SBN and LBN group and controls were used in the primary objective: to compare DNA methylation prior to NEC in children who developed NEC with DNA methylation in postnatal-age-matched controls, who did not develop NEC.

In the third group (After NEC, AN) we collected stool samples only after NEC development because these children had already developed NEC within a few days after birth. In this group stool samples from the preferred window around NEC onset were either not available or meconium-like. Meconium-like samples include intestinal cells that were shed during gestation and thus may not show a representative DNA methylation pattern. For these NEC infants we therefore chose to include the first available sample taken after the onset of NEC. DNA methylation changes of these samples may reflect ongoing NEC or NEC recovery instead of changes prior to NEC onset. These samples were only used in the secondary objective of the study, to assess the DNA methylation within NEC infants, at various time frames in relation to NEC. The selection of stool samples and the division in several time frames is displayed in Figure 1.

### DNA Isolation

The QIAamp Fast DNA stool mini kit (Qiagen, Hilden, Germany) was used according to the protocol to isolate DNA. From each stool sample, 220 mg of stool was used. An additional 30 s TissueLyser LT (Qiagen) step at 50 Hz with a stainless-steel bead was included at step 2 to obtain a homogenous stool sample. We checked quality and concentration of the isolated DNA with both the NanoDrop®ND-1000 spectrophotometer (Thermo Fisher Scientific, Waltham, MA) and by gel electrophoresis with 10 μl of the isolated DNA on 1% agarose gel with ethidium bromide staining. In case of a low DNA yield, DNA isolation was repeated using a different stool sample from the same infant, with a maximum of three repetitions per infant. We classified low DNA yield as <5 ng/μl of DNA on spectrophotometry or no DNA visible on gel electrophoresis. After DNA isolation, all samples underwent bisulfite treatment using the EZ DNA methylation Gold-Kit (Zymo Research, Irvine, CA) according to the supplier's protocol with an additional 30 s centrifuge round at full speed after step 8.

### Primer Design

The genomic target region selected for analysis was chosen based on relevant existing literature on gene expression and DNA methylation. DNA methylation occurs at cytosine-phosphate-guanosine (CpG) positions, therefore CpG rich areas in the promotor area were preferably selected. The analyzed promotor region for *EPO* includes the binding site for the hypoxia inducible factor (HIF) complex, which represses transcription through hypermethylation in several human cancers (26). The primers designed for *VEGFA* were designed to fit an area around a HIF binding site where hypomethylation is present in placental tissue following preeclampsia (27). For *ENOS*, the chosen site is part of a positive regulatory domain in the *ENOS* promotor, including binding sites for the transcriptional activators Sp-1/Sp-3 (28). Hypermethylation in this area is related to decreased activity and endothelial dysfunction (29). The promotor region selected for *DEFA5* is a CpG rich area in which hypermethylation and decreased expression is present in intestinal cells of Crohn's disease patients (30). The area selected for *TLR4* was chosen due to the presence of the binding sites for Sp-1 and regulatory factor X1, a *TLR4* transcription suppressor. The methylation status in this area is strongly associated with *TLR4* expression (31, 32). All PCR and sequencing primers for these target regions were designed using the Pyromark Assay Design software (Qiagen) and are presented in Table 1.

**Table 1:** PCR forward and reverse primer sequences accompanied by sequencing primer and the sequence to analyze, with its genomic region (Homo sapiens GRCh38.p12 primary assembly).

**Gene**	**Forward, reverse and sequencing primers**	**Sequence to analyze**	**Genomic region**
*EPO*	F: 5′-GGGGGTAGGGGT TGTTATTTGTATG-3′ R: 5′-Biotin-CCCAAACCTCCTAC CCCTACTCTAACC-3′ S: 5′-GGGTTGTTATTTGTATGTG-3′	TGYGTGYGYGGGTGGGGGTGGGG GAGAGGTTGTGTGYGTGAGGGG TYGTTAGGGGTAGGGGTT ATTYGGGGTTAGAGTAGGGGTAGGA	Chromosome 7 100720705–100720618
*VEGFA*	F: 5′-GGGAGTAGGAAAGTGAGGT-3′ R: 5′-Biotin-TTCCCCTACCCCCTTCAATAT-3′ S: 5′-AGTAGGAAAGTGAGGTTA-3′	YGTGYGGATAGGGT TTGAGAGTYGTTTTTTTT TTGTTAGGAATATTGA	Chromosome 6 43769901- 43769854
*DEFA5*	F: 5′-TAGGAGGTTGAGGTAGGAGAAA-3′ R: 5′-Biotin-ACATTATCCTTTAAT TCCATCCATATTATC-3′ S: 5′-GGAGGTTGTAGTGAGT-3′	YGGGATYGTATTATTGT ATTTTAGTTTGGGYGATA GTAAGATTTYGTTTTAAA AAAAAAAAAAAAAAAAA	Chromosome 8 7058585–7058654
*ENOS*	F: 5′-GTAGTGGGAGGGGGTTTT-3′ R: 5′-Biotin-ACCTCCCAACCCAA CTTATTCCTATCC-3′ S: 5′-GGGTTTTTTAGTGTTGGT-3′	TTTYGTTTYGT TTTTATTTTATAT ATAATGGGATAGGAAT AAGTTYGGTTGGGAGG	Chromosome 7 1050993691–1050993636
*TLR4*	F: 5′-GTTGAGGTTTATTTT TAGTTTTGTATGTG-3′ R: 5′-Biotin-AACCTCATTCTA CCTTACATACC-3′ S: 5′-GTGAGTTTTTTTATAAGAAGGG-3′	GYGGGTTAAATTGT GTTTTGTAAAAATTTAT ATATYGAAGTTTTAAT TTTTTTATTTTAGA	Chromosome 9 117703726–117703786

### PCR

For the DNA amplification we used 12.5 μl of Qiagen HotStar Taq Master Mix, 1 μl of 10 μM forward and reverse primer, 1 μl of bisulfite converted DNA and 10.5 μl purified water. Cycling conditions on the T100 Thermal Cycler (Bio-rad, Hercules, CA) were the same for all genes, except for *ENOS*: 95°C for 15 min, 50 cycles of 94°C for 30 s, 58°C (ENOS 56°C) for 30 s, 72°C for 30 s, followed by a final step of 72°C for 7 min and stored at 4°C. Due to the generally low yield of human DNA from stool samples, we optimized the PCR protocol to 50 cycles to increase the DNA amplification.

### Pyrosequencing

Pyrosequencing on a Pyromark Q48 system (Qiagen) was performed according to manufacturer's protocol followed by analysis using PyroMark Q48 Advanced Software (Qiagen). All samples were quality control checked and the percentage of DNA methylation at every CpG position calculated. Samples that failed quality control were excluded from data analysis.

### Statistical Analysis

Statistical analysis was performed using SPSS for Windows, version 23.0 (IBM Corp., Armonk, New York). Visual inspection of a Q-Q plot as well as a Shapiro-Wilk test were used to assess normality, with a *p*-value of <0.05 to be considered as non-normal distribution. Next, a parametric or non-parametric approach was chosen as appropriate. In case of a small sample size (*n* <5), we also chose a non-parametric approach. Continuous variables were assessed using a Mann-Whitney *U*, Kruskal-Wallis or Student's *T*-test. Categorical variables were analyzed using a Chi-Square test or a Fisher's Exact Test in case of an expected cell count below 5 and correlations were assessed using Pearson's Test or Spearman's Rho. Results with a *p* < 0.05 (two-tailed) were considered to be statistically significant. GraphPad Prism 7.02 was used to construct all figures.

## Results

This study cohort consisted of 24 infants with NEC and 48 controls. DNA isolation for three infants in the control group did not yield enough human DNA for further analysis. Therefore, the final control group consisted of 45 infants. The characteristics of the NEC infants and the control group are presented in Table 2. The median gestational age of the cohort was 27.4 weeks (IQR = 26.0–28.3) with a median birth weight of 920 grams (IQR = 780–1,075).

**Table 2:** Characteristics of the NEC infants and control group.

	**NEC**		**Control**		***P*-value**
	***N* = 24**		***N* = 45**	
Gestational age (weeks)	27.2	(25.6 to 28.2)	27.4	(26.0 to 28.4)	0.733^U^
Birth weight (grams)	900	(773 to 1,053)	950	(780 to 1,080)	0.450^x^
Birth weight (*z*-score)^a^	−0.95	(−1.64 to 0.10)	−0.57	(−1.29 to 0.04)	0.455^x^
Sex (male)	17	(71%)	27	(60%)	0.373^x^
Multiples	7	(29%)	13	(29%)	0.981^x^
SGA (< p10)	7	(29%)	12	(27%)	0.825^x^
Apgar score (5 min)	7	(6 to 8)	7	(6 to 8)	0.822^x^
Antibiotic exposure^b^	24	(100%)	44	(98%)	1.000^F^
Days	5	(2.5 to 11)	4	(2 to 8)	0.275^U^
Within 24 h (yes)	21	(88%)	38	(84%)	1.000^F^
Mother milk in the first 7 days	22	(92%)	43	(96%)	0.606^F^
NICU stay (days)	45	(22 to 58)	34	(28 to 53)	0.870^U^
Sepsis	3	(13%)	3	(7%)	0.344^F^
Mortality	7	(29%)	3	(7%)	0.011^x^
**Maternal**	***N*** **=** **23**		***N*** **=** **41**		
Primiparous	14	(61%)	26	(63%)	0.840^x^
Mode of delivery (CS)	9	(39%)	19	(46%)	0.577^x^
Preeclampsia or HELPP	4	(17%)	4	(10%)	0.443^F^
Antenatal steroids	22	(96%)	38	(93%)	1.000^F^
Antenatal antibiotics	12	(52%)	29	(73%)	0.103^x^
Placental abnormalities	18	(82%)	29	(78%)	0.729^F^
**Necrotizing enterocolitis**					
Age at onset NEC (days)	13	(9 to 25)	n.a.		
NEC as cause of death	6	(86%)	n.a.		
Bell stage IIA	14	(58%)	n.a.		
Bell stage IIB	3	(13%)	n.a.		
Bell stage III	7	(29%)	n.a.		
Treatment, conservative	15	(63%)	n.a.		
Treatment, surgical	9	(38%)	n.a.		

*Characteristics given as median and (IQR) or frequency and (percentage)*.

*SGA, small for gestational age, defined as a birth weight < p10; NICU, neonatal intensive care unit; CS, cesarean section; HELPP, Haemolysis Elevated Liver enzymes and Low Platelets syndrome; NEC, necrotizing enterocolitis; n.a., not applicable*.

a *According to Dutch Perined reference values (33)*.

b *Antibiotic exposure from birth until the date of the taken stool sample*.

U *Mann-Whitney U-Test*,

t *Students T-test*,

x *Chi-square Test*,

F *Fisher's Exact Test*.

### DNA Methylation Prior to NEC, Comparing NEC Infants With Controls

Samples reflecting methylation prior to NEC onset (SBN and LBN, *n* = 19) were taken a median of 2 days (IQR = 1–11) before NEC onset. Due to varying DNA quality, not all infants have DNA methylation results available for all five genes. The number of NEC infants and controls included for each gene as well as the mean DNA methylation percentages are presented in Table 3.

**Table 3:** Mean DNA methylation prior to NEC onset.

	**LBN**	**SBN**	**NEC (LBN + SBN)**	**Control**
*EPO*	n = 5	n = 14	n = 19	n = 40
Mean methylation	3.0 (2.4–3.2)	2.2 (2.0–2.8)	2.3 (2.0–2.9)	2.9 (2.0–4.0)
*VEGFA*	n = 5	n = 14	n = 19	n = 42
Mean methylation	1.6 (0.9–1.7)	1.6 (1.5–2.2)	1.6 (1.4–2.1)	1.7 (1.2–2.4)
*DEFA5*	n = 4	n = 9	n = 13	n = 26
Mean methylation	71.7 (69.6–73.9)	75.5 (72.6–77.6)	73.5 (72.2–76.3)	73.5 (71.9–77.1)
*ENOS*	n = 5	n = 13	n = 18	n = 39
Mean methylation	96.9 (95.8–100.0)	94.8 (92.2–98.7)	96.4 (92.2–98.9)	93.8 (87.5–99.0)
*TLR4*	n = 3	n = 12	n = 15	n = 36
Mean methylation	67.1 (66.1–78.6)	79.8 (76.5–84.2)	78.9 (75.8−84.2)	76.0 (67.7–83.0)

*Values shown as median and (IQR). NEC, Necrotizing Enterocolitis; LBN, Long Before NEC; SBN, a Short time Before NEC; NB: The LBN and SBN group were combined before comparison with the control group, and not compared separately with the control group in the prior to NEC analysis*.

**Figure 2:**
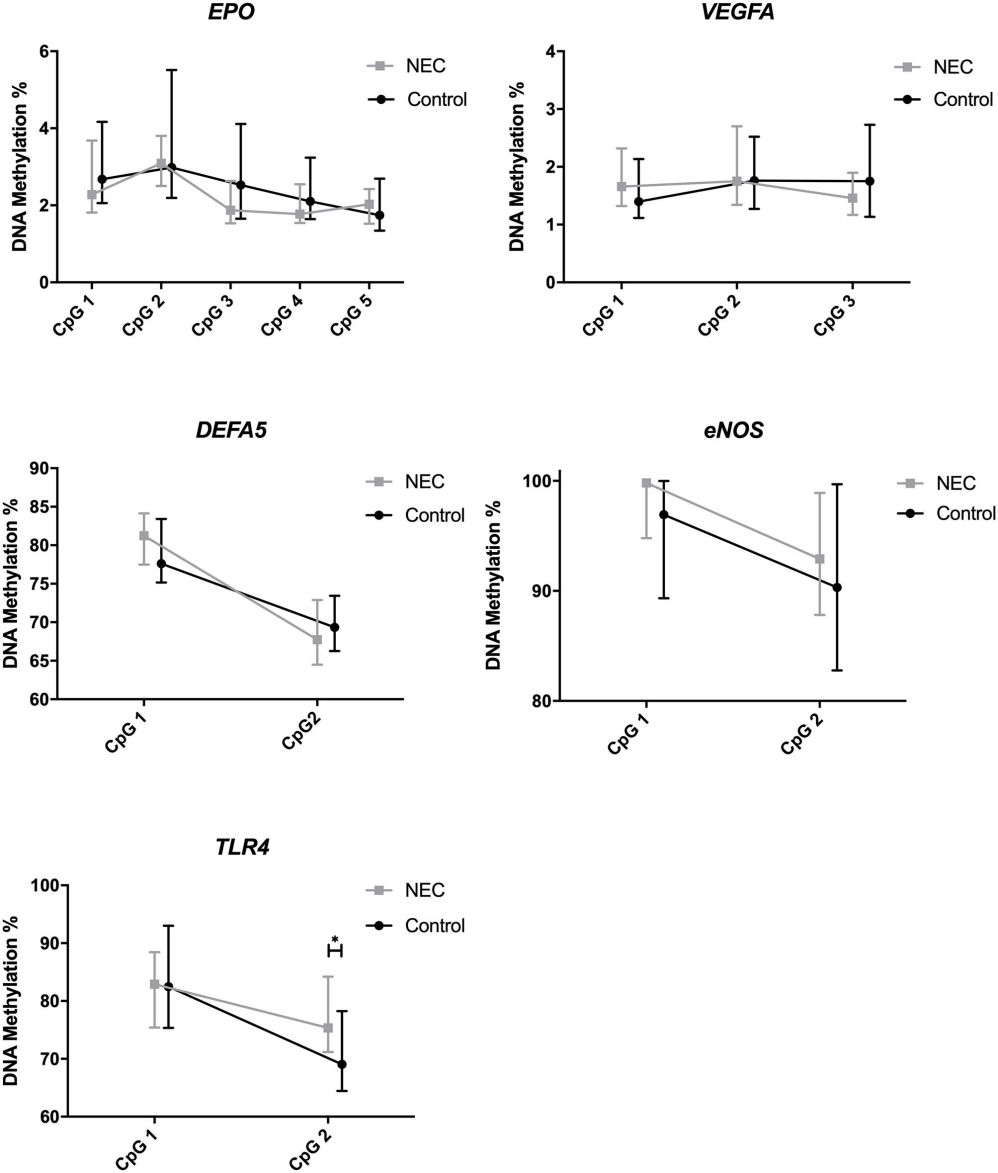
DNA methylation of *EPO, VEGFA, DEFA5, ENOS*, and *TLR4*, including individual CpG positions. Presented as median and IQR. * = *P* <0.05. NEC, Necrotizing Enterocolitis.

For the genes studied, we found a difference comparing the individual CpG positions of *TLR4*, which is involved in intestinal immunity through pattern recognition receptors, for NEC infants with controls. The DNA methylation of the individual CpG positions for all genes is displayed in Figure 2. *TLR4* CpG position 2 showed a significantly higher methylation (*p* = 0.025) in the NEC group (median = 75.4%, IQR = 71.3–83.8) than in the control group (median = 69.0%, IQR = 64.5–77.4). These differences did not lead to consistent changes in the mean methylation of the 2 CpG positions studied for *TLR4* between NEC infants and controls (Table 3). We noticed two outliers (>3 sd) in the control group. We repeated our analysis without these outliers and found similar results. No differences were found comparing the DNA methylation of *EPO, VEGFA, DEFA5*, and *ENOS* prior to NEC with controls.

### DNA Methylation in NEC Infants at Three Separate Time Frames

The characteristics of the NEC infants in the LBN, SBN, and AN group are presented in Table 4. NEC infants in the SBN group (*n* = 14) were significantly younger (median postnatal age = 12 days, IQR = 9–19) at the time of sample, compared with NEC infants in the LBN group (*n* = 5, median = 26 days, IQR = 24–27, *p* = 0.003) and the AN group (*n* = 5, median = 24 days, IQR = 21–27, *p* = 0.007).

**Table 4:** Characteristics of the NEC infants at three separate time frames.

**NEC groups**	**LBN (*n* = 5)**	**SBN (*n* = 14)**	**AN (*n* = 5)**
Gestational age (weeks)	26.0 (25.3–26.7)	27.2 (25.4–28.0)	28.4 (28.1–28.4)
Birth weight (grams)	875 (800–920)	885 (670−1,065)	1,040 (900–1,040)
Sex (male)	4 (80%)	10 (71%)	3 (60%)
Postnatal age at time of sample (days)	26 (24–27)	12 (9–19)	24 (21–27)
Time from sample to NEC onset (days)	14 (17–21)	1 (1–4)	10 (9–10)^∧^
Age at onset NEC (days)	41 (38–42)	11 (9–18)	9 (9–13)
Bell Stage IIA	4 (80%)	6 (43%)	4 (80%)
Bell Stage IIB	1 (20%)	2 (14%)	0 (0%)
Bell Stage III	0 (0%)	6 (43%)	1 (20%)
Treatment, conservative	5 (100%)	6 (43%)	4 (80%)
Treatment, surgical	0 (0%)	8 (57%)	1 (20%)
NEC as cause of death	n.a.	6 (43%)	n.a.

*Characteristics given as median and (IQR) or frequency and (percentage)*.

∧ *Days after NEC onset. NEC, Necrotizing Enterocolitis; LBN, Long Before NEC; SBN, a Short time Before NEC; AN, After NEC; n.a., not applicable*.

**Figure 3:**
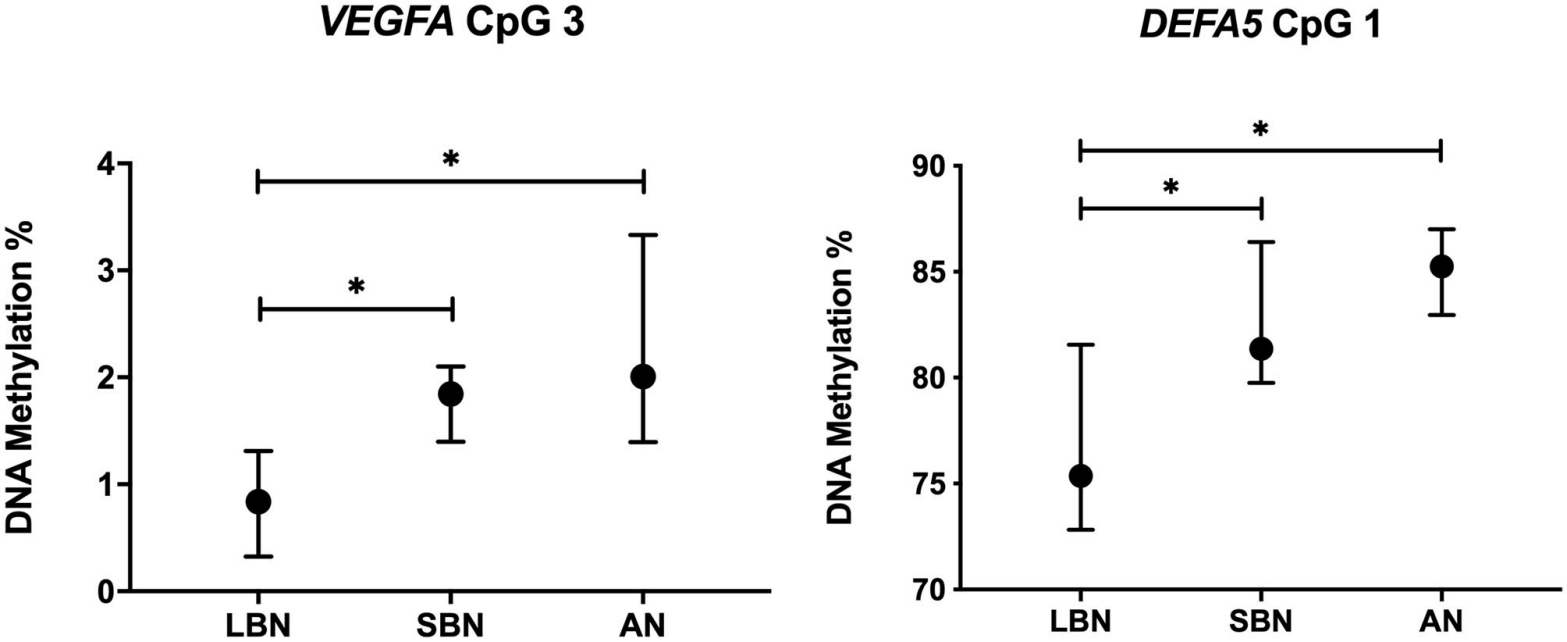
DNA methylation long before, a short time before and after NEC onset of *VEGFA* CpG 3 and *DEFA5* CpG 1. Presented as median and IQR. * = *P* <0.05. LBN, Long Before NEC, SBN, a Short time Before NEC; AN, After NEC; NEC, Necrotizing Enterocolitis.

We found a significant hypermethylation in the SBN and AN group for individual CpG positions of *VEGFA* and *DEFA5. VEGFA* is involved in intestinal perfusion, while *DEFA5* is involved in intestinal immunity. Figure 3 shows the DNA methylation of *VEGFA* CpG position 3 and *DEFA5* CpG position 1 in the three different groups. The number of infants included in each group as well as the DNA methylation percentages for all genes can be found in Supplementary Table 1.

Analyses of the individual CpG positions revealed a significant hypermethylation in *VEGFA* CpG 3 (*p* = 0.011) of infants in the SBN group (*n* = 14, median = 1.8%, IQR = 1.4–2.1) compared with infants in the LBN group (*n* = 5, median = 0.8%, IQR = 0.7–1.2). A significantly higher methylation (*p* = 0.021) was also found for infants in the AN group (*n* = 5, median = 2.0%, IQR = 1.5–2.1) compared with infants in the LBN group. Similarly, for *DEFA5* CpG 1, infants in the SBN group (*n* = 9, median = 81.4%, IQR = 79.9–84.8) showed a significantly higher methylation (*p* = 0.027) than infants in the LBN group (*n* = 4, median = 75.4%, IQR = 73.5–79.6). Comparing *DEFA5* CpG 1 for NEC infants in the AN group (*n* = 4, median = 85.3%, IQR = 83.4–86.8) with those in the LBN group also showed a significantly higher DNA methylation (*p* = 0.019).

Due to the selection procedure, postnatal age of the NEC infants differed between the three groups. We calculated the correlation coefficients between DNA methylation and postnatal age. DNA methylation for both *VEGFA* CpG 3 and *DEFA5* CpG 1 did not correlate with postnatal age (rho = −0.07, *p* = 0.74 and rho = −0.13, *p* = 0.63, respectively). We also calculated the correlation coefficients for DNA methylation of *VEGFA* CpG 3 and *DEFA5* CpG 1 and postnatal age in the control group. In this group, again DNA methylation for both *VEGFA* CpG 3 (*n* = 42) and *DEFA5* CpG 1 (*n* = 26) did not correlate with postnatal age (rho = 0.08, *p* = 0.61 and rho = 0.16, *p* = 0.44, respectively).

## Discussion

We compared DNA methylation of NEC infants with controls and found a hypermethylation in CpG position 2 of the *TLR4* gene in intestinal epithelial cells of preterm infants, prior to NEC onset. Furthermore, we found DNA methylation of *VEGFA* CpG 3 and *DEFA5* CpG 1 to be substantially higher in NEC infants a short time before and after NEC onset (SBN and AN) than long before NEC onset (LBN). We did neither find any differences in the DNA methylation of *EPO* and *ENOS* in NEC infants prior to NEC onset compared to controls, nor differences between the various time frames within the NEC group. The differences in DNA methylation of *TLR4, VEGFA*, and *DEFA5* may partially explain why some preterm infants with a similar risk profile develop NEC, while others do not. This exploratory study is the first to demonstrate that epigenetic changes such as DNA methylation early in life may contribute to this increased susceptibility. In the next sections we will discuss and interpret our findings in more detail, for each gene individually.

Regarding *TLR4*, we found a hypermethylation of CpG position 2 prior to NEC onset (LBN and SBN) when comparing NEC infants with controls. CpG position 2 is partially regulated by regulating factor X1, a potent transcription suppressor (32). A negative correlation between DNA methylation at CpG position 2 and mRNA expression of *TLR4* in CD14+ monocytes is reported in earlier studies (32). There, a decrease in DNA methylation of the non-affected group is associated with almost a doubling of TLR4 expression. The difference in methylation between both groups is around twice the size of the difference we found in the current study. Translating their findings to our own results, hypermethylation at CpG position 2 would still suggest a substantial decrease in the expression of *TLR4* in intestinal cells prior to NEC onset compared with controls. The downregulation of *TLR4* may cause an increased susceptibility for developing NEC, as it causes a weakened first line of defense against infiltration of pathogens, a well-known cornerstone of NEC development. Similar to our findings, a recent study shows a 13% increase of DNA methylation in the promotor region of TLR4 in surgically resected NEC colon (8). Here, TLR4 was one of several genes within the pathway of pattern recognition receptors that was hypermethylated compared to controls. While this hypermethylation suggests a downregulation of TLR4, no significant increase or decrease in TLR4 expression was reported.

Further evidence on *TLR4* methylation and expression in human tissue is limited. A higher expression of *TLR4* is present in human cell lines after NEC induction using experimental hypoxia and LPS (34). Furthermore, *TLR4* is expressed at higher levels in healthy preterm-born infants than in term-born infants and *TLR4* is assumed to be critical for fetal gut development *in utero* (35). Most of the evidence of *TLR4* involvement in NEC has been found in animal studies. The main findings include an increased expression of *TLR4* messenger RNA and protein in mice intestine after NEC induction and the inability to induce NEC in *TLR4* deficient mice (18, 20, 34, 36). The collective hypothesis of these studies suggests that the increased expression of *TLR4* causes an exaggerated *TLR4* activation and inflammatory response after bacterial colonization of the gut.

Concluding, *TLR4* is needed for mounting an adequate inflammatory response, therefore a decrease in *TLR4* might be detrimental because the inflammatory response might become inadequate, but on the other hand overactivation of *TLR4* might lead to an exaggerated inflammatory response inducing intestinal injury as seen in NEC. This “balancing-act” by *TLR4* needs further elucidation. Our findings suggest a downregulation in *TLR4* before NEC onset. Because of the lack of expression data in our study we can just urge that further studies into the regulation of *TLR4* regulation are necessary. Methylation changes in other areas or other concurrent epigenetic regulations, which were not evaluated here, may interfere with *TLR4* expression.

A second finding in this study was the hypermethylation, within our NEC infants, of *VEGFA* CpG position 3 and *DEFA5* CpG position 1 a short time before NEC onset and after NEC. Increasing methylation in *VEGFA* CpG position 3 is negatively associated with *VEGFA* expression (27). This is possibly due to the nearby binding site of hypoxia inducible factor 1A (HIF1A), a transcriptional factor known to suppress expression through hypermethylation (37). Similar to previous studies, the differences in DNA methylation percentages we found between groups were small. However, in previous studies a difference of several percentages is associated with an increase in *VEGFA* expression of 50–100% (27). Therefore, the hypermethylation of *VEGFA* CpG 3 we found in the period a short time before and after NEC suggests a reduced *VEGFA* expression due to NEC. Reduction in this angiogenic factor could result in an underdeveloped microvasculature of the intestine and poor perfusion a short time before onset and during or after NEC. Our findings are in line with results from experimental NEC models, in which a lower *VEGFA* expression is reported as well as a decrease in the VEGFA protein in intestinal tissue around NEC (14). We support the hypothesis that a decrease in *VEGFA* expression, possibly through DNA hypermethylation, increases the risk of developing NEC. Furthermore, continued downregulation of *VEGFA* after NEC onset may impair the recovery process.

Regarding *DEFA5*, we found similar results for its CpG position 1, with hypermethylation in the samples of our NEC group a short time before and after NEC onset compared with long before NEC onset. A negative relation between DNA methylation of the *DEFA5* promoter and its expression is reported in intestinal cells of Crohn patients (30). In this study, even small differences of several percentages in DNA methylation are associated with a 25–45% decrease in *DEFA5* expression. Extending these results to ours would imply that *DEFA5* expression is lowered from a short time before NEC onset onwards. A decreased expression would result in a lowered secretion of defensins, the antimicrobial agents produced by Paneth cells. This relative depletion in defensins could result in a weakened defense system, increasing the infant's risk for developing NEC. Currently, there are two contradicting hypotheses for the role of Paneth cells and *DEFA5* expression in NEC. The first hypothesis consists of an increased *DEFA5* expression, resulting in an increased inflammatory response as seen in NEC (38). Supporting this hypothesis is an increase of immune-competent Paneth cells and *DEFA5* expression starting at 29 weeks of gestation, corresponding with the peak incidence of NEC (38). The second hypothesis suggests a relative depletion or dysfunction of Paneth cells and a lowered *DEFA5* expression in NEC (39). This hypothesis is supported by animal studies using Paneth cell ablation to induce necrotizing enterocolitis in mice and the markedly diminished expression of defensins in preterm-born infants (39). Our results are more in line with this second hypothesis. In the inflamed intestinal cells of Crohn patients, a reduction of defensin expression is seen together with a hypermethylation of the promotor region of *DEFA5* (30). Here, the reduction of defensins is hypothesized to be a consequence of inflammatory changes, rather than the cause. It may be that a similar mechanism causes the hypermethylation we found in our study.

We did not find any differences in the DNA methylation of *EPO* and *ENOS*. For *EPO* we saw an overall low DNA methylation level, with little variation between subjects. If any differences between NEC infants and controls, or within the NEC group are present, it would probably require a larger sample size to detect these differences. We saw the opposite for *ENOS*, with generally high methylation levels, suggesting an overall low expression in intestinal cells. To detect any differences of *ENOS* methylation in NEC infants, possibly a cell type in which *ENOS* is expressed at a higher level should be used. Further studies should explore this.

The epigenetic changes we found may be related to environmental stressors known to be involved in NEC. For example, hypoxia can induce changes in DNA methylation of *VEGFA*, partially through hypoxia inducible factors, such as HIF1A (40). Furthermore, microbiota alterations are associated with differences in DNA methylation of *TLR4* and have also been linked with Paneth cell dysfunction (6, 41). As we did not evaluate these factors in the current study, we can only suggest their role in causing changes in DNA methylation.

An important additional finding and strength of this study is the novel non-invasive method we used to investigate the bowel of NEC infants. Until now, DNA analyses of stool samples of preterm infants were solely used for microbiome analysis. In this pilot study we extracted human DNA from intestinal cells in stool samples. The majority of fecal samples were taken right before NEC diagnosis, a median of 1 day before NEC onset in the largest group (SBN). This ensures a DNA methylation profile as close as possible to NEC onset. DNA methylation analysis by targeted pyrosequencing includes a sequence-specific PCR step, which precludes bacterial DNA from the analysis. Studies using human material for NEC research are limited and groups are often small. Collecting intestinal cells directly from the intestinal wall is only possible if an infant requires surgery. This occurs in around one third of NEC infants and virtually no healthy preterm infants, making it difficult to collect control group samples (42). A recent study used surgical samples in combination with laser capture microdissection, to measure epithelial cell-specific methylation (8). Their findings include a hypermethylation of TLR4 similar to our findings in this manuscript. This increases our confidence that the DNA methylation we measure is mainly from epithelial cells in stool. Using stool samples for DNA collection resulted in a relatively large and well-matched NEC-infant and control group in our study. While this study proves the use of stool samples as a non-invasive method for collecting human DNA in preterm NEC infants and controls, more research has to be done to further validate this method and determine the relation with gene expression.

We acknowledge several limitations of our study. First, it was not possible to include measurements of gene expression, as RNA is far less stable than DNA and degenerates quickly in stool samples. Therefore, we need to rely on literature for the extrapolation of our data to physiological function. Previous studies, however, have investigated the relation between methylation on the promotor regions of *TLR4, VEGFA*, and *DEFA5* and their gene expression. These studies all reveal a negative association between methylation and gene expression, which is in line with the general dogma that hypermethylation of promoter areas results in gene suppression. We therefore assumed this relation also existed in our cohort and that our findings are reflecting gene expression. In addition, we included several important CpG positions in the promotor region of each gene, but other CpG rich areas exist, in which similar or contrasting changes may occur. It is important to note that alongside DNA methylation, other epigenetic mechanisms such as histone modification and non-coding RNA regulate gene expression (43). We did not investigate these epigenetic mechanisms in this pilot study.

A second limitation concerns the low human DNA concentrations. Due to this we did not manage to successfully extract DNA from each stool sample, which resulted in some missing data. Furthermore, several outliers were present in all five genes, with methylation percentages very different in comparison to the rest of the group. We do not have indications that these were caused by analytical errors. As DNA methylation is known to be highly variable, we did not exclude these results from analysis. Even so, we also presented the results of the analyses excluding these outliers. A third limitation concerns multiple testing. As this was an exploratory study, we deliberately chose not to correct for multiple comparisons, acknowledging that some of our results may have been found by chance. In line with this, we did not perform multivariate testing due to the small sample size. Further larger prospective studies are warranted to confirm our results. Finally, we acknowledge that missing data may have biased our findings on the longitudinal part of our study, i.e., the changes of DNA methylation within the NEC infants, long before, a short time before and after NEC onset. We compared these three separate timeframes with varying sizes of samples and individuals. In addition, sample bias may unintentionally be present as a result of the variable onset of NEC in time. In future studies, prospectively and longitudinally collected stool samples of the same infants are required to confirm the change in DNA methylation over time that we found.

In conclusion, this is the first study to show differences in DNA methylation of *TLR4, VEGFA*, and *DEFA5* in infants affected by necrotizing enterocolitis. This partially supports our hypothesis that epigenetic modifications contribute to the increased susceptibility of some infants for developing NEC. Whether the observed differences are sufficient to alter gene expression and indeed are causally related to NEC should be explored in future studies.

## Data Availability Statement

The raw data supporting the conclusions of this article will be made available by the authors, without undue reservation.

## Ethics Statement

The studies involving human participants were reviewed and approved by Medical Ethical Review Board of the University Medical Center Groningen. Written informed consent to participate in this study was provided by the participants' legal guardian/next of kin.

## Author Contributions

DK, TP, EK, and AB conceptualized and designed the study. DK and RV-S collected the data. DK analyzed the data and drafted the initial manuscript. RV-S, TP, EK, and AB supervised the study. RV-S, TP, JH, EK, and AB reviewed and revised the manuscript critically. All authors approved the final manuscript as submitted.

## Conflict of Interest

The authors declare that the research was conducted in the absence of any commercial or financial relationships that could be construed as a potential conflict of interest.
