# Supplementary material for: DNA Methylation of TLR4, VEGFA, and DEFA5 Is Associated With Necrotizing Enterocolitis in Preterm Infants
Source: Front Pediatr. 2021 Mar 4;9:630817. doi: 10.3389/fped.2021.630817 (PMC7969816; doi:10.3389/fped.2021.630817)
Supplement: Supplementary file 1 [file Table_1.docx]

**Supplemental Table S1.** DNA methylation long before, a short time before and after NEC onset.

|  | LBN | SBN | AN | Control |
| --- | --- | --- | --- | --- |
| *EPO* | n = 5 | n = 14 | n = 5 | n = 40 |
| Mean methylation | 3.0 (2.4 – 3.2) | 2.2 (2.0 – 2.8) | 2.5 (2.4 – 5.7) | 2.9 (2.0 – 4.0) |
| CpG 1 | 2.3 (1.8 – 3.7) | 2.3 (1.9 – 3.3) | 2.2 (1.9 – 2.3) | 2.7 (2.1 – 4.2) |
| CpG 2 | 4.3 (4.0 – 6.0) | 2.8 (2.5 – 3.4) | 3.5 (2.0 – 4.1) | 3.0 (2.2 – 5.5) |
| CpG 3 | 2.2 (1.2 – 3.8) | 1.9 (1.6 – 2.5) | 2.9 (2.6 – 3.0) | 2.5 (1.7 – 3.9) |
| CpG 4 | 4.0 (1.8 – 5.9) | 1.6 (1.5 – 2.0) | 2.0 (1.9 – 2.0) | 2.1 (1.7 – 3.1) |
| CpG 5 | 2.3 (1.6 – 4.2) | 1.9 (1.5 – 2.2) | 1.6 (1.5 – 3.9) | 1.7 (1.4 – 2.7) |
| *VEGFA* | n = 5 | n = 14 | n = 5 | n = 42 |
| Mean methylation | 1.6 (0.9 – 1.7) | 1.6 (1.5 – 2.2) | 1.9 (1.7 – 2.1) | 1.7 (1.2 – 2.4) |
| CpG 1 | 1.7 (1.7 – 2.3) | 1.6 (1.3 – 2.2) | 2.0 (1.8 – 2.0) | 1.4 (1.1 – 2.0) |
| CpG 2 | 1.1 (1.0 – 1.8) | 2.0 (1.5 – 2.7) | 1.8 (1.6 – 1.9) | 1.8 (1.3 – 2.5) |
| CpG 3 | 0.8 (0.7 – 1.2) ^*†^ | 1.8 (1.4 – 2.1) ^*^ | 2.0 (1.5 – 2.1) ^*^ | 1.8 (1.2 – 2.7) |
| *DEFA5* | n = 4 | n = 9 | n = 4 | n = 26 |
| Mean methylation | 71.7 (69.6 – 73.9) | 75.5 (72.6 – 77.6) | 78.2 (77.8 – 78.6) | 73.5 (71.9 – 77.1) |
| CpG 1 | 75.4 (73.5 – 79.6) ^*†^ | 81.4 (79.9 – 84.8) ^*^ | 85.3 (83.4 – 86.8) ^†^ | 77.6 (75.3 – 83.1) |
| CpG 2 | 69.9 (61.4 – 72.4) | 67.7 (65.3 – 73.0) | 71.7 (69.5 – 73.2) | 69.3 (66.3 – 73.4) |
| *ENOS* | n = 5 | n = 13 | n = 5 | n = 39 |
| Mean methylation | 96.9 (95.8 – 100.0) | 94.8 (92.2 – 98.7) | 93.5 (93.4 – 97.4) | 93.8 (87.5 – 99.0) |
| CpG 1 | 100.0 (99.6 – 100.0 | 97.7 (95.0 – 100.0) | 100.0 (100.0 – 100.0) | 96.9 (89.4 – 100.0) |
| CpG 2 | 93.7 (92.1 – 100.0) | 89.6 (88.1 – 97.8) | 87.0 (86.7 – 94.9) | 90.3 (83.8 – 99.3) |
| *TLR4* | n = 3 | n = 12 | n = 5 | n = 37 |
| Mean methylation | 67.1 (66.1 – 78.6) | 79.8 (76.5 – 84.2) | 74.6 (67.7 – 77.5) | 76.0 (67.7 – 83.0) |
| CpG 1 | 62.0 (61.9 – 78.9) | 83.9 (78.9 – 88.0) | 76.6 (66.5 – 77.1) | 82.5 (75.6 – 92.7) |
| CpG 2 | 72.4 (70.4 – 78.4) | 75.5 (71.3 – 83.8) | 72.6 (68.9 – 77.8) | 69.0 (64.5 – 77.4) |
| Values shown as median and IQR. LBN = Long Before NEC, SBN = a Short time Before NEC, AN = After NEC. NEC = Necrotizing Enterocolitis. NB: The LBN, SBN and AN group were not compared separately with the control group.  * equals a p-value < 0.05 between LBN and SBN.  † equals a p-value <0.05 between LBN and AN. | | | | |
